# Clinical picture and treatment effects in 5 patients with Methylmalonic aciduria related to *MMAA* mutations

**DOI:** 10.1016/j.ymgmr.2019.100559

**Published:** 2020-01-08

**Authors:** Dorota Wesół-Kucharska, Magdalena Kaczor, Magdalena Pajdowska, Ewa Ehmke vel Emczyńska-Seliga, Anna Bogdańska, Dariusz Kozłowski, Dorota Piekutowska-Abramczuk, Elżbieta Ciara, Dariusz Rokicki

**Affiliations:** a Department of Pediatrics, Nutrition and Metabolic Diseases, The Children’s, Health Institute, Warsaw, Poland; b Department of Biochemistry, Radioimmunology and Experimental Medicine, The Children's Memorial Health Institute, Warsaw, Poland; c Department of Medical Genetics, The Children's Memorial Health Institute, Poland

**Keywords:** *MMAA*, CblA, Hydroxocobalamin, Renal failure

## Abstract

**Introduction:**

Methylmalonic Aciduria (MMA) is a heterogeneous group of rare diseases leading to accumulation of methylmalonic acid in body fluids. One of the causes of the disease is the methylmalonic aciduria, cblA type (*cblA* – type MMA), conditioned by a mutation in the *MMAA* gene, which is essential for the proper functioning of a cofactor of the methylmalonyl-CoA mutase. The symptoms of the disease, depending on the cause, may manifest themselves at different ages. Most patients are sensitive to high doses of hydroxycobalamin, which is associated with better prognosis.

**Material and method:**

The purpose of the study was to retrospectively analyze the clinical picture and effects of treatment of patients with methylmalonic aciduria related to mutation in the *MMAA* gene.

**Results:**

Five patients with diagnosed *cblA* – type MMA were presented. At the time of diagnosis the median of age was 18.8 months, but the symptoms had already appeared since infancy, as recurrent vomiting and delayed psychomotor development. Significant excretion of methylmalonic acid in urine and metabolic acidosis traits with significantly increased anionic gap were observed in all patients. All of them were sensitive to the treatment with vitamin B_12_. The median of therapy duration and observation is 12.2 years. During the treatment, good metabolic control was achieved in all patients, but their cognitive development is delayed. Three patients have renal failure and pharmacologically treated arterial hypertension.

**Conclusions:**

Patients with a mutation in the *MMAA* gene are sensitive to treatment with hydroxocobalamine, but the inclusion of appropriate treatment does not protect against neurodevelopmental disorders and chronic kidney disease.

## 1 Introduction

Methylmalonic aciduria (MMA) is a heterogeneous group of rare congenital metabolic abnormalities characterized by the accumulation of methylmalonic acid in body fluids and its excretion in urine. It results from disturbed metabolism/conversion of L-methylmalonyl-CoA to succinyl-CoA. This reaction depends on the mitochondrial enzymes: methylmalonyl-CoA epimerase and methylmalonyl-CoA mutase (MMUT), of which the cofactor is 5′-deoxyadenosine-cobalamin, corresponding to the *cblA, cblB* or *cblD-* subtypes, are caused by mutations in the *MMAA, MMAB,* or *MMADCH* gens, respectively [1]. The cofactor of MMUT requires cobalamin (vitamin B_12_) to work properly, therefore disorders of cobalamin absorption and transport (including vitamin B_12_ deficiency resulting from diet) may also be responsible for MMA. Deficiency of mitochondrial matrix enzymes (SUCLG1 and SUCLG2) are also responsible for isolated MMA [1,2].

Deficiency of MMUT is the most common, genetically determined cause of MMA (OMIM #251000). It is responsible for approx. 60% of isolated MMA cases and is most often conditioned by individual mutations [[3], [4], [5], [6]]. The disease variant associated with the deficiency of the MUT enzyme is divided into the so-called “mut^0^” (where the enzyme activity is indefinable) and “mut^−^” (related to its residual activity).

The *cblA* – type MMA deficiency is responsible for approx. 20% of causes of isolated MMA (OMIM #251100). It is conditioned by mutations in the *MMAA* gene, including the most frequently found change in the exon 2 (c.433C > T) [[7], [8], [9]]. So far, <200 patients with phenotype of *cblA*-type MMA have been described in the literature [6,7,[9], [10], [11]] .

In the “mut^0^” form, symptoms appear in the first days of a child's life, in the form of deterioration of the general condition with vomiting, aversion to eating, weight loss, apnea, thermoregulatory disorders and muscle tension. In laboratory tests, significant metabolic acidosis, hyperammomia, hyperketonuria, lactic acidosis, hypoglycaemia and hypertransaminasemia may occur [2,3,12]. Methylmalonic aciduria associated with residual enzyme activity (mut^−^ phenotype) and *cblA –* or *cblB* – type MMA manifests itself at a later age and has a rich but non-specific clinical presentation. In its course we observe encephalopathy, delay in psychomotor development, muscle hypotonia, seizures, symptoms of extrapyramidal syndrome and so called “metabolic” strokes in neuroimaging. In addition, there may be recurrent vomiting with ketoacidosis, anorexia, lack of normal weight gain and growth and occasional pancreatitis, as well as neutropenia or pancytopenia [[13], [14], [15]].

In the MMA process one of the most common organ complications is progressive chronic kidney disease, which occurs in the course of interstitial fibrosis with renal tubular atrophy. Nephrological problems are found in almost half of patients, which more often and at a younger age lead to kidney failure in patients with the phenotype of mut^0^ and *cblB* diseases, but may also occur in patients with *cblA* – type MMA or mut^−^ [10,16,17].

The diagnosis of MMA is based on biochemical tests. Increased production of methylmalonic acid and its metabolites is detected in urinary organic acid profile by gas chromatography (mass spectrometry, GC/MS) – methylmalonic acid, methylcytric acid, 3-hydroxypropionic acid are primarily detected, and acylocarnitine profile in dry blood spots by tandem mass spectrometry (tandem MS, MS/MS) – elevated levels of propionylcarnitine (C3-carnitine) and/or methylmalonylcarnitine (C4DC-carnitine) are found - this method is currently used in population screening of newborns in Poland [8,18]. These combined methods are able to provide a diagnosis of MMA, but not the underlying cause. For that, diagnosis must be made by mutation analysis and/or biochemical enzyme activity. Identification of the disease phenotype is important because forms with residual enzyme activity (mut^−^) and sensitivity to vitamin B_12_ (phenotype cblA, cblB, cblD2) are associated with better prognosis concerning the development and course of the disease [5,6,18].

The basic chronic management in patients with MMA is a diet with limitation of valine, isoleucine, methionine and threonine, supplemented by preparations with the so-called protein equivalent - free from “harmful” amino acids. In addition, the supply of hydroxocobalamin in high doses is recommended, but only in patients who respond to such a procedure. It is also recommended to supplement l-carnitine under control of its serum concentration and periodical decontamination of the digestive tract [2,18].

## 2 Objective of the study

The objective of the study was to evaluate the clinical course and treatment effects in patients with diagnosed cblA – type MMA.

## 3 Material and methods

A retrospective analysis of clinical course in patients of the Department of Pediatrics, Nutrition and Metabolic Diseases of CMHI in Warsaw with the diagnosed and molecularly confirmed methylmalonic aciduria resulting from *cblA*-type MMA, was performed. Molecular testing was conducted using a new generation sequencing method (NGS). Clinical status and abnormalities in additional examinations at the moment of diagnosis were analyzed. The current development, neurological disorders, as well as renal function parameters and metabolic equilibrium were evaluated. Delayed psychomotor development was defined as delay in reaching milestones later than 2 months (in the children under 2 years). Intellectual disability was assessed on the basis of standardized psychological tests and the following were defined IQ 85–70 - mild, IQ 69–50 - moderate, IQ below 50 - severe intellectual disability respectively. Each patient also had a diet analysis carried out by a clinical nutritionist.

## 4 Results

The clinical course of the disease in 5 patients (2 girls and 3 boys), including one pair of siblings, with diagnosed *cblA*-type MMA, was analyzed in this study. The median of the age at which the diagnosis was made is 18.8 months (4–54). At the time of diagnosis three children were <12 months old, 1 patient was >24 months old. The current median age of patients is 13.8 (6.4–18.7) years, and the median time of observation and treatment is 12.2 (5.2–17.8) years (Table 1). None of the patients had a detected disease in neonatal screening, as such screening for MMA in the Polish population has been carried out in selected provinces since 2009, and in the whole population since November 2013 (the youngest patient was born in 2011).

**Table 1:** Characteristics of the examined patients with cblA – type MMA.

Patient	1	2	3	4	5
Sex M/F (^⁎^ siblings)	F^⁎^	M^⁎^	F	M	M
Age at the time of diagnosis (months)	11	4	11	54	14
Current age (years)	18,6	15,8	14,5	13,8	6,4
*MMAA* mutation	c.590_593delTGAC	c.590_593delTGAC	c.266 T > C	c.590_593delTGAC	c.590_593delTGAC

Status at diagnosis					
Delayed psychomotor development	Yes	Yes	Yes	Yes	No
Disturbances of consciousness	Yes	No	Yes	Yes	No
Convulsions	No	No	Yes	No	No
Recurrent vomiting	Yes	Yes	Yes	Yes	Yes
Weight deficiency (<3 percentile for age and sex according to WHO)	Yes	Yes	No	Yes	No
Growth deficiency (<3 percentile for age and sex according to WHO)	No	No	Yes	Yes	No
Urinary methylmalonic acid (N < 20 mmol/mol creatinine)	2100	1500	1159	3180	2806
Serum ammonia (N: 20–80 μg/dl)	200	75	85	128	37
Increased activity of aminotransferases	No	Yes	Yes	Yes	No
Metabolic acidosis	Yes	Yes	Yes	Yes	Yes
Serum creatinine (N: 0,06–1,0 mg/dl)	0,54	0,31	0,30	1,84	0,28
Serum cystatin C (N: 0,5–0,96 mg/l)	0,93	0,73	0,78	2,11	0,98

Current status					
The number of metabolic decompensations since the start of treatment	0	0	4	0	2
Neurological condition	Mild intellectual disability, educational support no abnormalities on neurological exam, self-dependent	Mild intellectual disability, educational support no abnormalities on neurological exam, self-dependent	Severe intellectual disability, pyramidal extrapyramidal syndrome, requires help in everyday activities, fed by gastrostomy	Moderate, intellectual disability, special school, pyramidal extrapyramidal syndrome, wheelchair use	Mild intellectual disability no abnormalities on neurological exam, self-dependent
Weight deficiency (<3 percentile for age and sex according to WHO)	No	Yes	Yes	Yes	No
Growth deficiency (<3 percentile for age and sex according to WHO)	No	Yes	Yes	Yes	No
Renal failure (age of disclosure)	No	Yes (13 y)	No	Yes (4,5 y)	Yes (1,5 y)
Urinary methylmalonic acid levels (N < 20 mmol/mol creatinine)	210	293	100	74	73
Serum creatinine (N: 0,06–1,0 mg/dl)	0,68	0,67	0,48	0,58	0,69
Serum cystatin C (N: 0,5–0,96 mg/l)	0,93	1,0	0,99	1,73	1,47
eGFR^⁎⁎^ (N: > 75 ml/min/1,73 m^2^)	100,3	96,8	132,5	94,0	72,4
Low protein diet treatment	Yes	Yes	Yes	Yes	Yes
Hydroxocobalamin treatment	No	No	2 mg per week	3 mg per week	3 mg per week
L-karnityne treatment	1 g per day	1 g per day	0,5 g per day	1 g per day	0,5 g per day

N – norma, ^⁎⁎ ⁎^ the estimated Schwartz kidney filtration rate.

At the time of diagnosis, the majority of patients had delayed psychomotor development. Three children were in a severe general state with disturbances of consciousness, and one patient had convulsions. All patients had recurrent vomiting, 3 out of 5 patients had weight deficiency, including 2 patients with growth deficiency (<3 percentile for age and sex according to WHO).

In all patients, at the moment of diagnosis, the features of unbalanced metabolic acidosis with significantly increased anionic gap were found, but only in two patients the concentration of ammonia was mild increased. In all patients significant excretion of methylmalonic acid in urine was found using GC/MS method. In 3/5 there was detected an increased activity of aminotransferases. In addition, more than half of the patients had anemia, hyperglycemia and ketonuria. In one patient elevated serum creatinine and in two serum cysyatin concentration was observed. At the time of diagnosis, 4/5 of the patients had cortical and subcortical atrophy and ventricular dilatation in neuroimaging studies.

After balancing the general condition and introducing a diet with limited valine, isoleucine, threonine and methionine, a trial with vitamin B_12_ (intramuscular supply of hydroxocobalamin 1 mg) showed a decrease in the excretion of methylmalonic acid in urine (Table 2), which confirms the clinical sensitivity to hydroxocobalmine. The test with hydroxocobalamin was carried out after the introduction of the diet and reduced urinary excretion of MMA due to difficulties in access to the drug - in Poland only cyanocobalamin is routinely available.

**Table 2:** MMA excretion after intake of vitamin B12 intramuscularly. Significantly lower at the beginning of the test MMA excretion than previous diagnosis was influenced by previously introduced dietary treatment.

Patient number	1	2	3	4	5
MMA excretion before vitamin B12 intramuscularly injection	317	218	254	760	420
MMA excretion 1 day after intake of vitamin B12 intramuscularly (mmol/mol creatinine)	265	180	nd	nd	280
MMA excretion 2 days after intake of vitamin B12 intramuscularly (mmol/mol creatinine)	280	155	150	307	120
MMA excretion 3 days after intake of vitamin B12 intramuscularly (mmol/mol creatinine)	180	150	100	170	98

nd – no data available.

Molecular tests showed mutations in the *MMAA* gene in all patients (Table 1).

Currently, 3 patients receive hydroxocobalmine (intramuscular injections 2–3 times a week - 1 mg), 2 patients do not tolerate this type of treatment. From the moment of diagnosis all the patients remain on a diet with limited isoleucine, valine, methionine and threonine and with l-carnitine supplementation, under the control of a clinical dietician. During the treatment, acute metabolic decompensations requiring hospitalization and treatment were observed several times (2 and 4 times respectively) in only 2 patients.

Currently, biochemical parameters of metabolic control in all patients remain normal: none of the patients have metabolic acidosis, elevated concentration of lactic acid or hyperamonemia. High excretion of methylmalonic acid in urine: median 155 mmol/mol creatinine (*N* < 20) is still observed in all patients. In 3 out of 5 patients chronic kidney disease was diagnosed. The diagnosis was made at the age of 13–4.5 and 1.5 years, respectively, on the basis of cystatin C concentration. Only one patient had elevated creatinine concentration (1.84 mg/dl) and decreased eGFR (29.3–37 ml/min/1.73 m^2^) at the time of diagnosis. At present, normal renal function is maintained in all patients: the estimated Schwartz kidney filtration rate (eGFR) is <75 ml/min/1.72 m^2^ in only 1 patient, creatinine concentration in all patients remains within normal limits, but cystatin C concentration is elevated in 3 patients (Table 1). Arterial hypertension, well controlled pharmacologically, occurs in 3/5 patients. No cardiomyopathy or pancreatitis has been diagnosed so far.

Three patients have weight and height deficiency (<3 percentile for age and gender according to WHO centile grids). All patients have a diagnosed intellectual disability; in addition, two patients present features of pyramidal extrapyramidal syndrome (spastic limb paresis, dystonic movements and choreatetosis). In 4 patients in whom magnetic resonance imaging of the head was performed, cortical and subcortical atrophy, brain ventricular dilatation, and additionally in one of them a signal increase in T2-dependent images in both pallidal nuclei was observed.

## 5 Discussion

Mutation in the *MMAA* gene responsible cblA – type MMA is related to a mild phenotypic methylmalonic aciduria. In most cases, it is associated with a response to hydroxocobalmine (parenteral supply of vitamin B12 causes a significant reduction in serum methylmalonic acid concentration and its excretion in urine), which directly translates into better prognosis [1,5,17,19,20]. Presumably, after the introduction of appropriate treatment (diet, vitamin B_12_, l-carnitine), the patients presented rarely metabolic decompensations, and at the time of the study no metabolic imbalance features were found. All patients are alive to the present day.

Early diagnosis of MMA in neonatal screening and the inclusion of appropriate treatment is associated with better prognosis of patient development and less acute metabolic decompensation, which may be a life-threatening condition [3,10,18]. Presented patients were not included in screening tests, and the diagnosis was established after infancy on the basis of GC/MS profile of organic acids in urine and acylocarnitine profile in MS/MS in dry blood droplets. At present, in Poland it is not possible to determine the activity of enzymes responsible for MMA, which is not a prerequisite for making a diagnosis. The diagnosis should be confirmed by a molecular examination, and it is necessary to determine the cause of MMA [17,18].

All patients from the moment of diagnosis remain on a diet with limited natural protein, under the control of a clinical dietician. They also receive l-carnitine. Clinically, sensitivity to hydroxocobalamine was also demonstrated in all patients. Nevertheless, the development of the presented patients is not satisfactory. This may result from late diagnosis and late implementation of treatment, as well as the already presented delay in psychomotor development at the moment of diagnosis. Patients with *ClbA* – type MMA have better psychomotor development compared to patients with MMA due to a deficit of MMUT (especially mut^0^ phenotype) and *cblB* – type MMA [4,7,10,17,19]. Changes identified in neuroimaging in the presented patients are similar to those presented in the literature, including the most frequently described atrophy of the cerebral cortex, brain ventricular dilatation and peri-chamber myelinization disorders [21].

Even with sensitivity to vitamin B_12_ and compliance with dietary recommendations, the most common complication in patients with MMA is chronic kidney disease (CKD), which occurs in less than half of the patients with a median age of 6.5–7.5 years. The progression to end-stage disease requiring renal replacement therapy occurred in 12–14% of patients [3,10]. In patients sensitive to vitamin B_12_ CKD it was found less frequently in comparison to patients without response to treatment with hydroxocobalmine (16% vs 36%), and in patients with mut^0^ phenotype it reached even 55% [3]. In the presented patients renal failure was assessed in 3 patients who also had arterial hypertension. Complications resulting from chronic kidney disease (hypertension, anemia, renal osteodystrophy and hyperparathyroidism) aggravate the basic disease [10,22,23].

The treatment of patients with MMA and CKD does not differ from the standard treatment of renal failure for other reasons [18]. However, it is problematic to determine renal function. The glomerular filtration rate (GFR) based on serum creatinine concentration may be falsified due to weight loss (low muscle mass), which often occurs in patients with MMA, and due to a low protein diet. It is proposed that in MMA patients renal function should be assessed on the basis of cystatin C and concentration of methylmalonic acid in serum [18]. Increased excretion of methylmalonic acid in urine correlates with the risk of CKD, but with the development of renal failure it ceases to be a reliable marker and its serum concentration should therefore be determined [13,16,22,24].

An alternative therapy to the standard treatment of patients with MMA is liver and/or kidney transplantation. It is particularly recommended to consider this option in patients with frequent metabolic decompensation and, of course, in patients with extreme renal failure requiring renal replacement therapy [2,[23], [24], [25], [26]].
